# Effect of polyethylene glycol loxenatide on weight loss in super-obese patients with type 2 diabetes: a randomized controlled trial

**DOI:** 10.3389/fendo.2026.1689040

**Published:** 2026-01-30

**Authors:** Zenglin Liu, Changrong Song, Yunlu Cai, Zhewen Li, Sanyuan Hu

**Affiliations:** 1 Department of General Surgery, Shandong Provincial Qianfoshan Hospital, Shandong University, Cheeloo College of Medicine, Jinan, Shandong, China; 2 Department of General Surgery, The First Affiliated Hospital of Shandong First Medical University and Shandong Provincial Qianfoshan Hospital, Jinan, Shandong, China; 3 Department of General Surgery, Qilu Hospital, Cheeloo College of Medicine, Shandong University, Jinan, Shandong, China

**Keywords:** adverse reactions, glucagon-likepeptide-1 receptor agonist, glycemic control, polyethylene glycol loxenatide, super obesity, type 2 diabetes mellitus, weight loss

## Abstract

**Objective:**

To investigate the efficacy and safety of polyethylene glycol loxenatide (PEG-Loxe) for weight reduction and metabolic improvement in super-obese patients with type 2 diabetes mellitus (T2DM).

**Method:**

This was a single-center, single-blind, randomized, controlled clinical trial. A total of 123 study participants were enrolled and randomly assigned to the PEG-Loxe low-dose group (300 μg/week), PEG-Loxe high-dose group (400 μg/week), and placebo group. In total, 105 participants completed the study. The primary endpoint was the difference in weight reduction between groups.

**Results:**

All three groups had similar baseline characteristics. The least squares mean, 95% confidence interval (LSM, 95% CI) change in weight from baseline to week 24 was greater in the PEG-Loxe 300 μg group -16.34 (-19.10, -13.58) kg and PEG-Loxe 400 μg group -21.14 (-23.90, -18.37) kg compared to the placebo group -6.75 (-9.51, -3.99) kg (*P* < 0.001). Glycated hemoglobin levels decreased from the baseline by 0.50%, 1.02%, and 1.34% in the placebo, low-dose, and high-dose groups, respectively. The PEG-Loxe treatment group showed a significant reduction in waist circumference and increased high-density lipoprotein levels during the study period. The rate of adverse drug reactions was higher in the PEG-Loxe 400 μg group (36.11%) and the PEG-Loxe 300 μg group (25.00%) than in the placebo group (11.43%). Adverse reactions were predominantly gastrointestinal.

**Conclusion:**

For super-obese patients with T2DM, continuous treatment with PEG-Loxe for 24 weeks can effectively reduce body weight and achieve good glycemic control.

**Clinical Trial Registration:**

www.chictr.org.cn, identifier ChiCTR2100052922.

## 1 Introduction

Type 2 diabetes mellitus (T2DM) is associated with overweight and obesity and its prevalence is rapidly increasing worldwide, particularly in developing countries (1). A 2010 study reported 92 million adults with diabetes in China (2). More than half of these individuals are overweight or obese (3). Being overweight and obese are direct risk factors for type 2 diabetes (4), especially abdominal obesity, which promotes insulin resistance, increases the prevalence of prediabetes and diabetes (5, 6), and is associated with adverse clinical outcomes (7).

The prevalence of obesity has continued to increase (8), with an estimated 52% increase within 5 years (9). Obesity is directly associated with a significant increase in the prevalence of metabolism-related diseases including diabetes. Moreover, as the degree of obesity increases, the incidence of metabolism-related diseases gradually increases. Obesity with a body mass index (BMI) exceeding 50 kg/m² is termed super obesity (10), and relatively few metabolism-related treatment studies have targeted this population. Due to the severity of obesity, limited mobility, and poor compliance, it is difficult to achieve effective metabolic improvement and weight control through lifestyle interventions alone (11). Research has indicated that weight loss in overweight or obese type 2 diabetes patients is associated with reduced insulin resistance and improved glycemic control indicators (12), making weight reduction an important treatment goal for patients with T2DM (13). Therefore, for a special population with super-obesity, especially those with concurrent type 2 diabetes, it is imperative to find suitable treatment methods to control blood glucose levels while effectively reducing weight and improving metabolism.

Glucagon-like peptide-1 (GLP-1) is an incretin hormone primarily secreted by intestinal L cells. Following food intake, GLP-1 regulates glucose and lipid metabolism via multiple pathways, inducing insulin secretion and reducing glucagon secretion in a glucose-dependent manner (14). In addition to glucose reduction, it can enhance islet function (15). GLP-1 also delays gastric emptying and induces satiety, leading to decreased energy intake and weight loss.

GLP-1 receptor agonists (GLP-1RAs) are a class of medications that mimic the action of GLP-1 secreted by the human intestinal tract, having significant clinical value in improving glucose metabolism and weight management. Currently, some GLP-1RAs such as liraglutide and semaglutide are marketed for blood glucose control and weight reduction. PEG-Loxe is a long-acting GLP-1RA that was approved for clinical use in China in 2019 for blood glucose control. Compared with short-acting GLP-1 receptor agonists such as liraglutide and benaglutide, its mature commercial product requires only once-weekly injections, which helps improve medication adherence. Previous studies have shown that weight reduction was observed in non-obese patients with T2DM using PEG-Loxe (16–18).It suggests that in obese patients with type 2 diabetes, it may help control blood glucose levels while also reducing body weight. Currently, there are no studies on the effects of PEG-Loxe on weight reduction, glucose reduction, or metabolic improvement in super-obese patients with T2DM.

## 2 Materials and methods

### 2.1 Trial design and participants

This single-center, single-blind, randomized controlled clinical trial was conducted in accordance with the Declaration of Helsinki and was approved by the Ethics Committee of the First Affiliated Hospital of Shandong First Medical University (IRB: YXLL-KY-2021 (044)). The study was registered in the Chinese Clinical Trial Registry (Identifier: ChiCTR2100052922), and written informed consent was obtained from all study participants. Designed and completed this research report according to Consolidated Standards of Reporting Trials. From January 2022 to June 2024, 123 participants were enrolled in the study, of whom 105 ultimately completed it. The inclusion criteria for study participants were: (1) age 18–65 years, (2) BMI ≥ 50 kg/m², (3) diagnosis of type 2 diabetes according to the 1999 World Health Organization criteria, and (4) provision of written informed consent. The exclusion criteria were: (1) type 1 diabetes, (2) intellectual disability or mental illness, (3) morbid obesity with obvious precipitating factors, (4) pregnancy or lactation, (5) use of other GLP-1 receptor agonists or dipeptidyl peptidase-4 inhibitors within the past 3 months, (6) contraindications to trial-related medications, (7) allergy to trial-related medications, (8) weight change exceeding 10% within the past 3 months, (9) history of gastrointestinal disease or pancreatitis, and (10) participation in conflicting research protocols.

### 2.2 Procedures

The study included a 4-week run-in period and 24-week treatment period for a total of 28 weeks. Participants with super obesity and T2DM who had poor glycemic control despite lifestyle interventions were recruited. During the run-in period, participants underwent diabetes assessment at the diabetes outpatient clinic, where standardized lifestyle intervention measures were established and maintained throughout the study period. Participants who met the inclusion criteria were enrolled and started oral metformin treatment under the guidance of an endocrinologist. The metformin dosing protocol was standardized, with an initial dose of 500 mg/day. During the run-in period, the dose was increased to 500 mg weekly and escalating from 500 mg/day to 1500 mg/day. The maintenance dose was 1500 mg/day, and the metformin dose remained unchanged throughout the treatment period.

At the end of the run-in period, participants who demonstrated good tolerability to metformin and satisfactory adherence were enrolled in the final study. All the enrolled participants underwent baseline assessments at the same hospital, followed by a 24-week treatment period with group assignments. Eligible participants were randomized in a 1:1:1 ratio into a placebo group, a PEG-Loxe 300 μg group, or a PEG-Loxe 400 μg group. Randomization was performed using a computer-generated sequence. Group allocations were concealed in sequentially numbered opaque envelopes prepared by an independent staff member, which were opened only after a participant completed the run-in period and was eligible for assignment, thereby ensuring allocation concealment. The placebo or study drug was administered via subcutaneous injection into the abdominal area once a week for 24 weeks. The drug is a colorless and transparent liquid. In this study, saline was used as the placebo, administered with identical syringes at the same injection sites. This ensured that study participants could not distinguish between the placebo and the drug based on injection appearance or discomfort, thereby maintaining the blinding procedure. This study was single-blind, and blinding was applied only to the participants. In addition, the data statisticians were blinded to the group assignments during data analysis. Participants underwent standardized blood glucose monitoring in accordance with diabetes clinic requirements and returned to the hospital weekly for data collection, standardized dosing, and safety assessments. Demographic data, height, weight, blood pressure, waist circumference, hip circumference, and laboratory test results were also collected. Laboratory tests (conducted every 8 weeks) included glycated hemoglobin (HbA1c), fasting plasma glucose (FPG), fasting insulin, lipid profile, and liver function. An oral glucose tolerance test (OGTT) was performed every 12 weeks.

### 2.3 Endpoints

The end of the 24-week drug treatment period marked the end of the study. The primary endpoint was the difference in weight change from baseline between the different groups. Secondary endpoints include the magnitude of weight change across different groups; the proportion of participants achieving weight reductions of 5%, 10%, and 15%; the degree of HbA1c reduction after treatment; the proportion of participants with HbA1c ≤ 6.5% and HbA1c <7.0%; and improvements from baseline in FPG, 2-h postprandial glucose (PPG), waist circumference reduction, total cholesterol (TC), triglycerides (TG), low-density lipoprotein cholesterol (LDL-C), high-density lipoprotein cholesterol (HDL-C), and blood pressure. The safety endpoints included the incidence of adverse events (AE), adverse reactions, and serious adverse events.

### 2.4 Statistical analysis

The 114 participants planned for the study were randomly assigned to receive PEG-Loxe or placebo. This sample size was expected to provide 80% power to detect a difference of ≥ 5% in total body weight loss between the PEG-Loxe group and the control group, with a standard deviation (SD) of 4.25% (19), an α level of 0.05, and a dropout rate of 20%. This estimate is based on data from previous study evaluating the weight-loss effects of PEG-Loxe in overweight patients with T2DM. We recognize that in individuals with super obesity, extremely high baseline body weight may lead to greater variability; however, the actual differences in weight reduction observed between groups in this trial substantially exceeded 5%, and the statistical power was adequate.

Statistical analyses were performed using IBM SPSS Statistics for Windows, version 25.0. P < 0.05 was considered statistically significant. Normality of the data distribution was assessed using the Shapiro–Wilk test. For quantitative data with normal distribution, results are presented as mean ± standard deviation. Baseline data across the three groups were compared using one-way analysis of variance (ANOVA). Paired t-tests were used to analyze differences before and after treatment within each group. Analysis of covariance (ANCOVA) was used to evaluate the differences in efficacy among the three groups before and after treatment, with the randomized treatment group as the factor variable and the corresponding baseline data as covariates. For data that did not conform to a normal distribution, the results were presented as medians (upper and lower quartiles). Comparisons between two groups were performed using non-parametric rank-sum tests (Mann–Whitney U test), whereas comparisons among multiple groups were performed using the Kruskal–Wallis H test. For count data, results were presented as frequencies and rates (or composition ratios), and intergroup comparisons were performed using chi-square tests. Bonferroni correction was applied for multiple comparisons of percentage of total weight loss (TWL%), percentage of excess weight loss (EWL%), HbA1c, FPG, 2-hour postprandial glucose (2h-PPG), waist circumference (WC), triglycerides (TG), cholesterol (TC), high-density lipoprotein cholesterol (HDL-C), low-density lipoprotein cholesterol (LDL-C), systolic blood pressure (SBP), diastolic blood pressure (DBP), alanine aminotransferase (ALT), and aspartate aminotransferase (AST). The efficacy analysis was conducted on an intention-to-treat (ITT) basis. No protocol deviations in the study. Multiple linear imputations were used for missing data. Safety analysis was conducted using the safety set (SS), which included study participants who used the study drug and had at least one safety evaluation record.

## 3 Results

**Figure 1:**
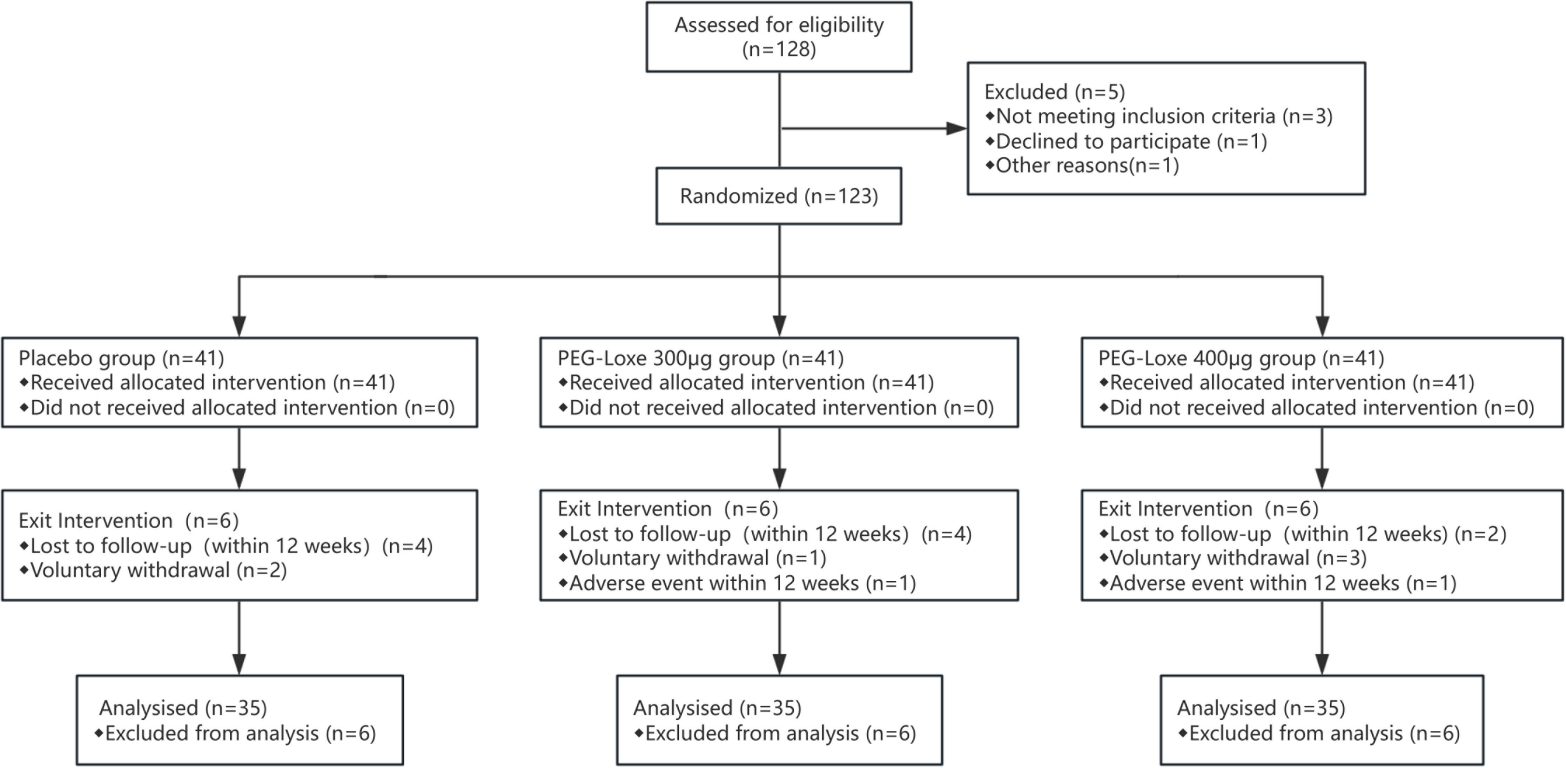
Consort flow diagram.

From January 2022 to June 2024, 123 participants were enrolled and randomized into a placebo group, a PEG-Loxe 300 μg group, and a PEG-Loxe 400 μg group. In total, 105 participants completed the study and were followed up. Six participants voluntarily withdrew from the study, and 12 withdrew due to AEs and loss to follow-up (**Figure 1**).

The demographic and baseline characteristics of the PEG-Loxe 300 μg group, PEG-Loxe 400 μg group, and placebo group are shown in **Table 1**. No significant differences were observed between the three groups of study participants in terms of sex ratio, age, weight, HbA1c, FPG, fasting insulin, blood lipids, waist circumference, blood pressure, ALT, AST, or other parameters.

**Table 1:** Baseline characteristics of patients.

Physiological indicators	Placebo (n=35)	PEG-Loxe 300µg (n=35)	PEG-Loxe 400µg (n=35)	statistics	*P value*
Male (n/%)	22 (62.9%)	24 (68.6%)	25 (71.4%)	0.609^1^	0.738
Female (n/%)	13 (37.1%)	11 (31.4%)	10 (28.6%)		
Age (years)	31.95 ± 7.88	33.77 ± 8.23	29.49 ± 7.03	2.707^2^	0.072
Weight (kg)	169.09 ± 31.00	168.63 ± 24.98	170.71 ± 20.09	0.064^2^	0.938
BMI (kg/m^2^)[median (IQR)]	53.34 (51.20-59.56)	54.01 (51.21-56.47)	54.13 (52.07-58.82)	1.332^3^	0.514
WC (cm) [median (IQR)]	152 (144-170)	152 (147-159)	154 (150-162)	1.307^3^	0.520
HbA1C (%)[median (IQR)]	7.6 (6.8-8.5)	7.2 (6.5-9.7)	7.1 (6.7-8.1)	0.531^2^	0.767
FPG (mmol/L)[median (IQR)]	7.49 (6.29-9.32)	7.34 (5.91-10.22)	7.67 (6.78-9.24)	0.724^3^	0.647
FPI (μU/ml)	37.21 ± 17.72	37.55 ± 38.88	38.02 ± 25.72	0.007^2^	0.993
2h-PPG (mmol/L)[median (IQR)]	14.59 (11.47-16.55)	12.98 (8.95-19.75)	14.25 (11.57-17.56)	0.715^3^	0.672
HOMA-IR	12.67 ± 6.05	12.16 ± 10.22	13.93 ± 10.15	0.358^2^	0.700
TG (mmol/L)	2.12 ± 1.22	1.74 ± 0.79	1.94 ± 1.14	1.126^2^	0.328
TC (mmol/L)	4.88 ± 0.93	4.64 ± 0.99	4.68 ± 0.77	0.682^2^	0.508
HDL-C (mmol/L)	1.03 ± 0.18	1.11 ± 0.18	1.09 ± 0.20	1.478^2^	0.233
LDL-C (mmol/L)	3.00 ± 0.85	2.78 ± 0.76	2.98 ± 0.71	0.864^2^	0.425
SBP (mmHg)	139.20 ± 15.16	142.00 ± 16.71	143.40 ± 18.32	0.582^2^	0.568
DBP (mmHg)	88.51 ± 13.20	89.17 ± 12.24	90.63 ± 15.23	0.221^2^	0.802
ALT (U/L)[median (IQR)]	36.6 (25.9-48.1)	26.3 (17.9-71.0)	52.4 (23.2-85.6)	3.939^3^	0.140
AST (U/L)	28.49 ± 14.96	28.27 ± 19.82	34.39 ± 21.93	1.153^2^	0.320

BMI, body mass index; WC, waist circumference; HbA1C, glycated hemoglobin; FBG, fasting blood glucose; FPI, Fasting Plasma Insulin; PPG, postprandial blood glucose; HOMA-IR, homeostatic model assessment of insulin resistance; TG, triglycerides; TC, cholesterol; HDL-C, high-density lipoprotein cholesterol; LDL-C, low-density lipoprotein cholesterol; SBP, systolic blood pressure; DBP, diastolic blood pressure; ALT, alanine aminotransferase; AST, aspartate aminotransferase; IQR, interquartile range. Data are presented as mean ± standard deviation unless otherwise specified. ^1^ indicates the *χ*² value; ^2^ indicates the *F* value; ^3^ indicates the *H* value; *P*-value denotes comparisons among the three groups.

### 3.1 Primary endpoint

Participants in all three groups experienced varying degrees of weight loss during the study period. The two PEG-Loxe treatment groups achieved a significantly greater weight reduction (*P* < 0.001), with no significant differences between the dose groups. At week 24, the least squares mean (LSM, 95% CI) change from baseline in body weight was −16.34 (−19.10, −13.58) kg for the PEG-Loxe 300 μg group, −21.14 (−23.90, −18.37) kg for the PEG-Loxe 400 μg group, and −6.75 (−9.51, −3.99) kg for the placebo group. Specifically, PEG-Loxe 300 μg group led to an additional 9.59 kg weight loss (95% CI: 4.80–14.38 kg) compared to placebo, and PEG-Loxe 400 μg group led to 14.39 kg more weight loss (95% CI: 9.60–19.18 kg) versus placebo group, both *P* < 0.001 (**Table 2**).

**Table 2:** Differences in weight changes among the three groups.

Variable		Placebo	PEG-Loxe 300µg	PEG-Loxe 400µg
		(n=35)	(n=35)	(n=35)
Body weight (kg), mean ± SD				
baseline		169.09 ± 31.00	168.63 ± 24.98	170.71 ± 20.09
After 24 weeks		162.37 ± 29.02	152.43 ± 22.71	149.37 ± 18.86
Intra-group differences	*t*	5.553	9.865	13.565
	*p*	<0.001	<0.001	<0.001
Change of Body Weight at 24 weeks (kg)				
LSM (95% CI)		-6.75 (-9.51, -3.99)	-16.34 (-19.10, -13.58) ^a^	-21.14 (-23.90, -18.37) ^a^
*vs*. placebo		–	9.59 (4.80,14.38)	14.39 (9.60,19.18)

SD, standard deviation; LSM: least squares mean; CI, confidence interval; ^a^ indicates comparison with the placebo group, *P* < 0.001.

### 3.2 Secondary endpoints

**Figure 2:**
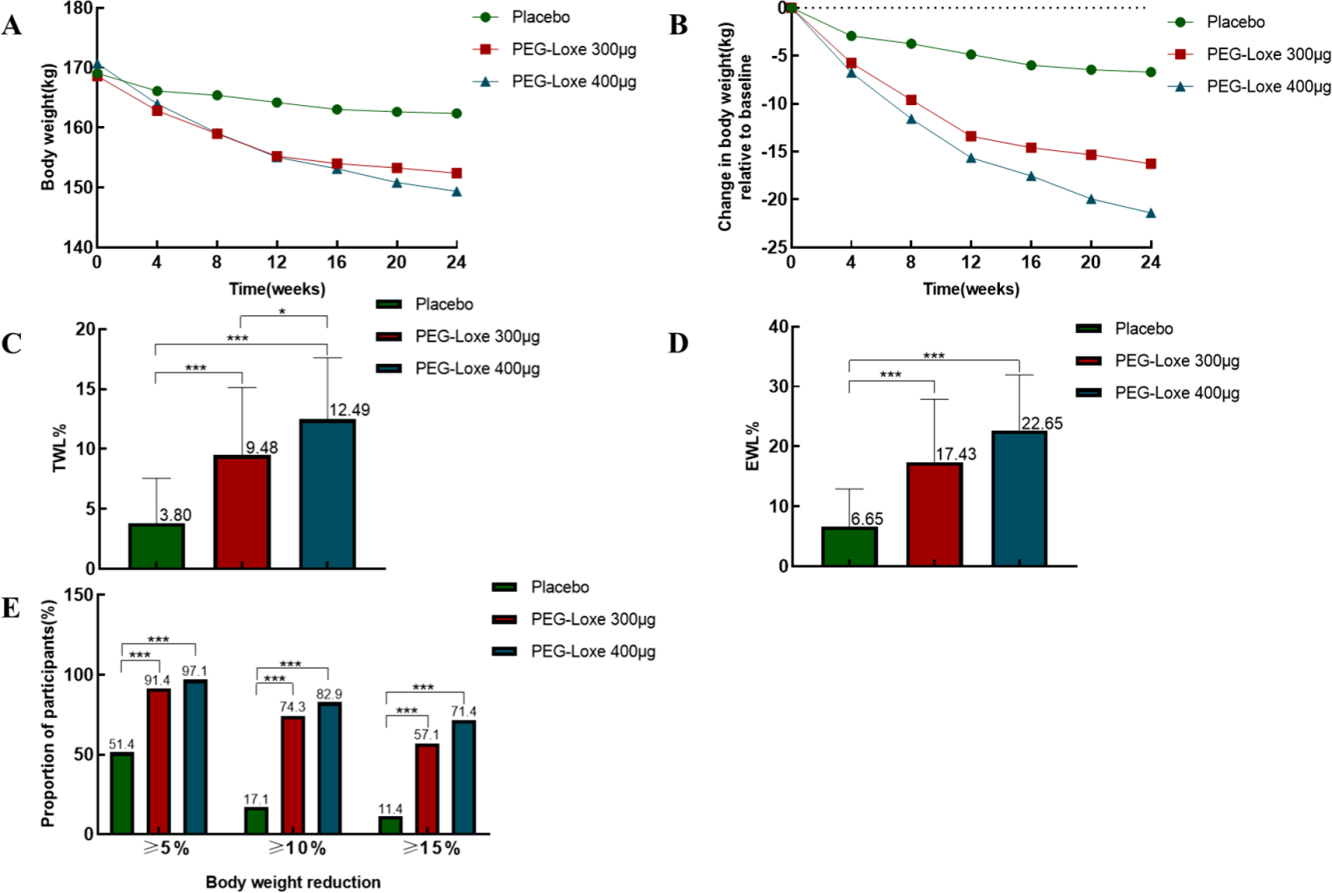
Differences among the three groups in terms of weight loss. **(A)**: Weight change curves of the three groups during the 24-week study period. **(B)**: Weight change curves relative to baseline for the three groups during the 24-week study period. **(C)**: Total weight loss percentage for the three groups after 24 weeks. **(D)**: Additional weight loss percentage for the three groups after 24 weeks; **(E)**: Proportion of participants in the three groups achieving 5%, 10%, and 15% weight loss after 24 weeks. *P<0.05,***P<0.001. %TWL: Percentage of total weight loss; %TWL = (initial body weight - final body weight)/initial weight × 100%. %EWL: Percentage of excess weight loss; %EWL = [(initial body weight - final body weight)/(initial weight - ideal body weight)] × 100%. Ideal BMI (IBMI): 25 kg/m^2^.

As shown in **Figures 2A, B**, all three groups experienced substantial weight loss during the first 12 weeks, with the rate of weight reduction slowing after 12 weeks. At the end of the 24-week period, both the PEG-Loxe 300 µg group and PEG-Loxe 400 µg group demonstrated superior performance compared to the placebo group in terms of total weight loss and additional weight reduction (**Figures 2C, D**), with statistically significant differences (*P* < 0.001). The proportions of participants achieving greater than 5% total weight loss were 54.1%, 91.4%, and 97.1% for the placebo group, PEG-Loxe 300 µg group, and PEG-Loxe 400 µg group, respectively. The proportions that achieved > 10% weight loss were 17.1%, 74.3%, and 82.9%, respectively. Among the PEG-Loxe 300 µg and PEG-Loxe 400 µg groups, 57.1% and 71.4% of study participants achieved >15% total weight loss, respectively, compared to 11.45% in the placebo group.

**Figure 3:**
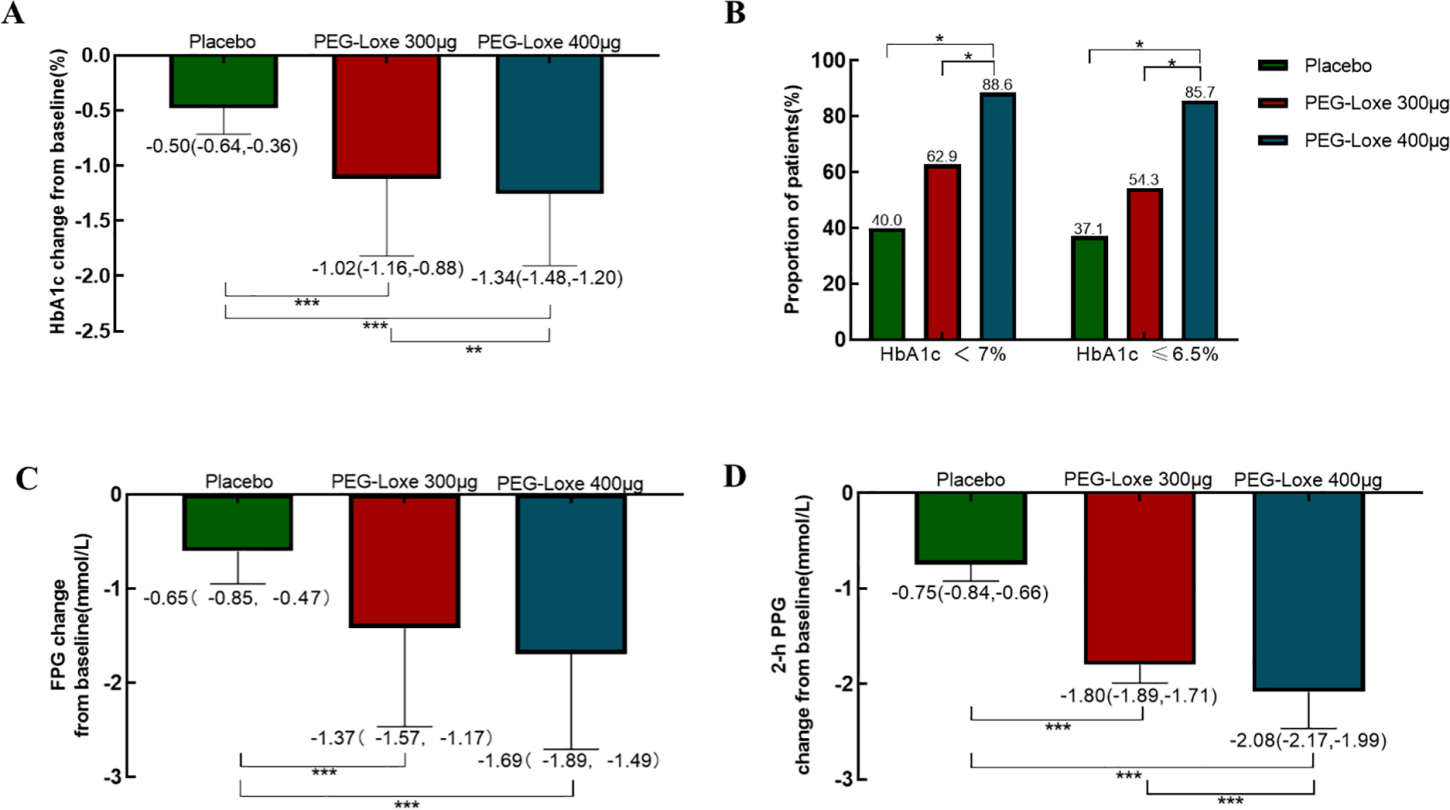
Changes in blood glucose at 24 weeks. **(A)**: Change curve of glycated hemoglobin (HbA1c) from baseline at 24 weeks. **(B)**: Proportion of subjects with HbA1c < 7.0% and HbA1c ≤ 6.5% at 24 weeks. **(C, D)**: LSM (95% CI) changes in fasting plasma glucose (FPG) and 2-hour postprandial glucose (2h-PPG) between baseline and week 24. *P<0.05,**P<0.01,***P<0.001.

Regarding glycemic control, as shown in **Figure 3**, the LSM (95% CI) changes in HbA1c from baseline to week 24 were more pronounced in the PEG-Loxe 300 μg group at -1.02% (-1.16%, -0.88%) and the PEG-Loxe 400 μg group at -1.34% (-1.48%, -1.20%) (**Figure 3A**). Overall, both PEG-Loxe doses achieved superior HbA1c reduction compared to the placebo (*P* < 0.001). At week 24, the PEG-Loxe 400 μg group demonstrated greater HbA1c reduction than the PEG-Loxe 300 μg group (*P* < 0.01). As illustrated in **Figure 3B**, the proportions of patients achieving HbA1c < 7% were 40.0%, 62.9%, and 88.6% for the placebo group, PEG-Loxe 300 μg group, and PEG-Loxe 400 μg group, respectively, while the proportions of patients with HbA1c ≤ 6.5% were 37.1%, 54.3%, and 85.7%, respectively. The LSM (95% CI) changes in FPG from baseline to week 24 were significantly greater (*P* < 0.001) in both PEG-Loxe groups compared to placebo group: -1.37 (-1.57, -1.17) mmol/L and -1.69 (-1.89, -1.49) mmol/L for the PEG-Loxe 400 μg group and 300 μg group, respectively, versus -0.65 (-0.85, -0.47) mmol/L in the placebo group (**Figure 3C**). At week 24, the changes in 2h-PPG from baseline were -1.80 (-1.89, -1.17) mmol/L and -2.08 (-2.17, -1.99) mmol/L for the PEG-Loxe 300 μg group and 400 μg group, respectively, both significantly greater (*P* < 0.001) than the placebo group at -0.75 (-0.84, -0.66) mmol/L (**Figure 3D**).

As shown in **Table 3**, at the end of week 24, all three groups of study participants showed significant reductions in waist circumference compared to baseline, with both the PEG-Loxe 300 µg group and PEG-Loxe 400 µg group demonstrating greater changes than the placebo group (*P* < 0.05). High-density lipoprotein levels in the PEG-Loxe 300 µg and PEG-Loxe 400 µg groups increased by 0.23 (0.16, 0.30) mmol/L and 0.37 (0.30, 0.44) mmol/L respectively compared to baseline, showing significant differences compared to the placebo group at -0.07 (-0.14, 0.01) mmol/L (*P* < 0.001). No significant differences were observed between the three groups regarding changes in TG, TC, LDL-C, systolic blood pressure (SBP), diastolic blood pressure (DBP), alanine aminotransferase (ALT), or aspartate aminotransferase (AST) levels from baseline.

**Table 3:** Changes in waist circumference, blood lipids, blood pressure, ALT, and AST from baseline at 24 weeks.

Physiological indicators	Placebo (n=35)	PEG-Loxe 300µg (n=35)	PEG-Loxe 400µg (n=35)	*P* value
WC (cm)	-3.44 (-5.27, -1.61)	-8.29 (-10.12, -6.46) ^c^	-9.33 (-11.14, -7.52) ^a^	<0.05
TG (mmol/L)	-0.22 (-0.60,0.16)	0.16 (-0.22,0.54)	0.11 (-0.26,0.49)	0.312
TC (mmol/L)	-0.28 (-0.61,0.06)	-0.34 (-0.68, -0.01)	-0.06 (-0.39,0.28)	0.452
HDL-C (mmol/L)	-0.07 (-0.14,0.01)	0.23 (0.16,0.30) ^a,b^	0.37 (0.30,0.44) ^a^	<0.001
LDL-C (mmol/L)	-0.01 (-0.25,0.23)	-0.30 (-0.54, -0.06)	-0.3 (-0.54, -0.06)	0.160
SBP (mmHg)	-0.79 (-7.64,6.17)	0.48 (-6.32,7.29)	-0.74 (-7.58,6.10)	0.957
DBP (mmHg)	-2.48 (-7.10,2.13)	-2.97 (-7.56,1.61)	-4.40 (-9.00,0.19)	0.832
ALT (U/L)	-13.88 (-24.57, -3.19)	2.50 (-8.15,13.15)	-16.95 (-27.71, -6.18)	0.260
AST (U/L)	-7.51 (-13.40, -1.61)	-0.98 (-6.88,4.92)	-8.11 (-14.03, -2.17)	0.177

Data are expressed as LSM (95% CI). WC, waist circumference; TG, triglycerides; TC, cholesterol; HDL-C, high-density lipoprotein cholesterol; LDL-C, low-density lipoprotein cholesterol; SBP, systolic blood pressure; DBP, diastolic blood pressure; ALT, alanine aminotransferase; AST, aspartate aminotransferase; ^a^ indicates comparison with the placebo group, *P* < 0.001; ^b^ indicates comparison with the PEG-Loxe 400 µg group, *P* < 0.05; ^c^ indicates comparison with the placebo group, *P* < 0.01.

### 3.3 Safety evaluation

Using the safety analysis set for drug safety evaluation, the incidence rates of AEs in the placebo group, PEG-Loxe 300 µg group, and PEG-Loxe 400 µg group were 11.43% (4/35), 25.00% (9/36), and 36.11% (13/36), respectively (**Table 4**). The incidence of adverse reactions in both drug groups was higher than that in the placebo group (*P* < 0.05), with one study participant in each group withdrawing from the study because of adverse reactions. The remaining study dropouts were due to loss to follow-up or withdrawal of consent rather than drug-related intolerance. This study conducted follow-ups with participants using standardized questionnaires to identify potential adverse reactions, and performed medical assessments and management for any adverse reactions that occurred. (**Supplementary Table S1**) Gastrointestinal reactions were the most common adverse reactions, and the incidence of gastrointestinal reactions increased in a dose-dependent manner with increasing PEG-Loxe dosage, with symptoms occurring predominantly within the first 4 weeks of treatment. No serious hypoglycemic events or deaths occurred during the study period.

**Table 4:** Summary of safety.

AEs (n) %	Placebo (n=35)	PEG-Loxe 300µg (n=36)	PEG-Loxe 400µg (n=36)
Any AE	4/35 (11.43%)	9/36 (25.00%)	13/36 (36.11%)
Severe	4/35 (11.43%)	7/36 (19.44%)	11/36 (30.56%)
Moderate	0/35 (0.00%)	2/36 (5.56%)	1/36 (2.78%)
Mild	0/35 (0.00%)	0/36 (0.00%)	1/36 (2.78%)
Discontinuation because of AEs	0/35 (0.00%)	1/36 (2.78%)	1/36 (2.78%)
Nausea	1/35 (2.86%)	3/36 (8.33%)	7/36 (19.44%)
Vomiting	0/35 (0.00%)	1/36 (2.78%)	2/36 (5.56%)
Decreased appetite	0/35 (0.00%)	1/36 (2.78%)	3/36 (8.33%)
Abdominal bloating	0/35 (0.00%)	2/36 (5.56%)	3/36 (8.33%)
Diarrhea	2/35 (5.71%)	0/36 (0.00%)	1/36 (2.78%)
Injection site discomfort	1/35 (2.86%)	1/36 (2.78%)	1/36 (2.78%)
Dizziness	0/35 (0.00%)	0/36 (0.00%)	1/36 (2.78%)
Headache	0/35 (0.00%)	1/36 (2.78%)	0/36 (0.00%)
Fatigue	0/35 (0.00%)	0/36 (0.00%)	1/36 (2.78%)
Hypoglycemia	0/35 (0.00%)	0/36 (0.00%)	0/36 (0.00%)

AE(s), Adverse Event(s).

## 4 Discussion

GLP-1 is a natural incretin hormone secreted by intestinal L-cells in response to food intake. Its core function is glucose-dependent glycemic regulation, which promotes insulin secretion, inhibits glucagon secretion, delays gastric emptying, and enhances satiety. Natural GLP-1 is rapidly degraded in the body and has a short half-life (20). GLP-1 RAs are a class of drugs developed based on the mechanism of action of GLP-1, and have become a revolutionary breakthrough in the field of T2DM treatment. By mimicking the mechanism of action of natural GLP-1 in the human body, these drugs exert therapeutic effects; influence glucose and lipid metabolism; and regulate appetite, nutrient absorption, and gastrointestinal motility (20). In recent years, related drugs have provided significant weight reduction and metabolic benefits and some products have already been approved for managing obesity (21). PEG-Loxe belongs to the long-acting GLP-1 RAs category, requiring only once-weekly administration to meet the glycemic regulation requirements, which is beneficial for improving medication adherence. Multiple studies on PEG-Loxe glycemic regulation and improvement of metabolism-related diseases have been conducted (19, 22, 23). However, research on the drug’s efficacy and safety regarding weight reduction and metabolic improvement in super-obese populations is currently limited. And no direct comparisons with other GLP-1RAs have been made, so any potential advantages of PEG-Loxe remain to be confirmed. This study focuses solely on evaluating the efficacy and safety of PEG-Loxe in weight reduction and blood glucose lowering among patients with super obesity.

Obesity is a chronic metabolic disease characterized by abnormal glucose and lipid metabolism and is associated with the occurrence and development of multiple diseases (24). It significantly increases the risk of developing T2DM, fatty liver disease, cardiovascular disease, and various cancers (25). The goals of treating obesity and related metabolic disorders are to reduce body weight, improve metabolic parameters, and lower the risk of cardiovascular disease (26). Previous studies have shown that GLP-1 RAs are promising pharmaceutical options to meet obesity treatment requirements (27). In addition to medications, bariatric surgery is currently a safe and effective treatment method for patients with morbid obesity and related conditions, offering advantages in achieving long-term sustained weight loss, addressing medical problems associated with severe obesity, improving quality of life, and reducing mortality rates. Super obesity refers to an extreme form of obesity with a BMI ≥ 50 kg/m². It may be accompanied by insulin resistance and disorders of glucose and lipid metabolism, while also increasing the risk of hypertension, cardiovascular disease, sleep apnea–hypopnea syndrome, fatty liver, and certain cancers. Moreover, morbid obesity is significantly associated with an increased risk of mortality (28). For super-obese patients, lifestyle interventions alone are insufficient to achieve effective weight loss and glycemic improvement; therefore, bariatric surgery remains the most effective treatment approach for this population (11). However, superobese patients face high surgical risks, making preoperative weight intervention advisable (29). Effective preoperative weight reduction is crucial for perioperative safety and satisfactory surgical outcomes in patients with super-obesity. Preoperative weight loss and glycemic control in obese patients are important components of preoperative management in bariatric surgery protocols, as they can improve surgical prognosis and reduce the morbidity and mortality associated with bariatric surgery. Even a modest preoperative weight loss of 5% can significantly reduce related risks (30). In this population, pharmaceutical treatment alone may be insufficient to achieve and maintain highly satisfactory weight loss. Bariatric surgery can induce substantially greater weight loss in obese patients (about 24% of initial body weight within approximately 24 weeks) (31), far exceeding the 10%–13% total weight loss achieved with PEG-Loxe in 24 weeks. However, pharmacotherapy can still play a critical preoperative role. In our study, PEG-Loxe enabled the majority of patients to lose >10% body weight – well above the 5% weight loss known to significantly improve surgical safety and outcomes. This suggests that PEG-Loxe could be utilized as a preoperative weight reduction strategy to lower surgical risk in super-obese T2DM patients who are candidates for bariatric surgery.

Weight reduction is one of the advantages of GLP-1 RAs, and a weight loss of 5%–10% or more is significantly associated with improvement in obesity-related metabolic diseases, including T2DM (32). In terms of weight reduction, GLP-1 RAs treatment is more effective than standardized lifestyle interventions alone (33). This study aimed to evaluate the efficacy and safety of PEG-Loxe for weight reduction and metabolic improvement in super obese populations with T2DM, with the study design including two drug groups of 300 μg and 400 μg doses. A total of 105 study participants with a mean age of 31.73 ± 7.86 years completed this study, of whom 67.6% were male and 32.4% were female, with a mean weight of 169.47 ± 25.51 kg. Drug treatment was continued for 24 weeks, and both the PEG-Loxe treatment groups achieved satisfactory primary efficacy endpoints. At 24 weeks, the PEG-Loxe 300 μg and 400 μg groups showed mean reductions of 10.44% and 13.49% of total body weight from baseline, respectively, exceeding the placebo group’s 3.92% (*P* < 0.001). The proportions achieving the weight control targets of >5% weight loss were 91.4% and 97.1%, respectively, whereas the proportions achieving >10% weight loss were 74.3% and 82.9%, respectively, with weight reduction persisting throughout the study period.

GLP-1 RAs demonstrate excellent glycemic control effects. In this study, both PEG-Loxe groups showed highly significant improvements in blood glucose levels. After 24 weeks of treatment, the PEG-Loxe 300 μg group achieved an average HbA1c reduction of 1.02%, the 400 μg group achieved a reduction of 1.34%, and the placebo group showed a reduction of 0.50%. These results are consistent with previous PEG-Loxe-related clinical trial outcomes (17, 34). Furthermore, at week 24, the proportion of participants achieving the improvement targets of HbA1c < 7.0% and HbA1c ≤ 6.5% was higher than in the placebo group. Another advantage of long-acting GLP-1 RAs is their ability to effectively reduce FPG and PPG levels (35). The PEG-Loxe 300 μg and 400 μg groups and placebo group showed mean reductions in FPG from baseline of 1.37 mmol/L, 1.69 mmol/L, and 0.65 mmol/L, respectively, while 2h-PPG decreased by -1.80 mmol/L, -2.08 mmol/L, and 0.75 mmol/L, respectively, demonstrating a dose-dependent relationship. The use of GLP-1 RAs also improves obesity-related metabolic diseases, such as cardiovascular and cerebrovascular diseases, hypertension, and hyperlipidemia (36). In this study, the drug groups showed reduced waist circumference and increased HDL-C levels, indicating an improvement in body fat distribution and lipid profile. A reduction in waist circumference suggests a decrease in visceral adiposity, which is associated with improved insulin sensitivity and lower cardiovascular risk. The rise in HDL cholesterol, albeit modest, is also clinically favorable, as higher HDL-C is protective against cardiovascular disease in obesity and diabetes.

Regarding adverse reactions related to GLP-1 RAs treatment, both Astrum and Mehta et al. reported adverse reactions during treatment with related drugs, with gastrointestinal symptoms being the most common (37, 38). In this study, the adverse reactions observed in the PEG-Loxe treatment groups were similarly characterized by mild gastrointestinal symptoms, primarily occurring within the first 4 weeks after initial administration. All symptoms resolved spontaneously within a short period and no severe hypoglycemic events occurred in any of the three groups. The incidence of adverse reactions was 25.00% in the PEG-Loxe 300 µg group and 36.11% in the PEG-Loxe 400 µg group, both higher than the 11.43% observed in the placebo group. Because all three groups in this study received concomitant metformin treatment, the gastrointestinal adverse reactions in each group could not be entirely attributed to PEG-Loxe use alone. Future studies investigating PEG-Loxe monotherapy are required to explore the incidence of drug-related adverse reactions.

This study was conducted at a single center with a relatively small sample size. Multicenter prospective studies with longer follow-up periods are still needed to evaluate the long-term efficacy of the drug, as well as dedicated PEG-Loxe monotherapy trial designs, to explore the effects of single-agent treatment on weight reduction and metabolic improvement in superobese populations. Individuals with super obesity typically present with higher baseline body weight, more complex metabolic profiles, and greater potential for weight reduction. Consequently, their response to interventions may differ from that of the general obese population, with a greater magnitude of expected weight loss. The findings may not be generalizable to overweight or obese individuals with lower body weight; further subgroup analyses from similar studies are required to provide more robust validation and minimize these limitations. Additionally, this trial was single-blind (only participants blinded), which could introduce observer bias in outcome measurements by investigators. We acknowledge that investigator awareness of treatment allocation might have subtly influenced measurements and adverse event classification. This design, along with the single-center setting, limits the generalizability of our findings and could bias the results.

## 5 Conclusion

In patients with super obesity complicated byT2DM, PEG-Loxe effectively reduced body weight and controlled blood glucose levels during the 24-week treatment period, demonstrating good drug safety. However, further clinical trials are required to determine its long-term therapeutic effects and the duration of effect maintenance after drug discontinuation.

## Data Availability

The raw data supporting the conclusions of this article will be made available by the authors, without undue reservation.

## References

[1] Chen L Magliano DJ Zimmet PZ . The worldwide epidemiology of type 2 diabetes mellitus–present and future perspectives. Nat Rev Endocrinol. (2011) 8:228–36. doi: 10.1038/nrendo.2011.183, PMID: 22064493

[2] Yang W Lu J Weng J Jia W Ji L Xiao J . Prevalence of diabetes among men and women in China. N Engl J Med. (2010) 362:1090–101. doi: 10.1056/NEJMoa0908292, PMID: 20335585

[3] Ji L Hu D Pan C Weng J Huo Y Ma C . Primacy of the 3B approach to control risk factors for cardiovascular disease in type 2 diabetes patients. Am J Med. (2013) 126:925.e11–.e22. doi: 10.1016/j.amjmed.2013.02.035, PMID: 23810406

[4] Kadowaki T Isendahl J Khalid U Lee SY Nishida T Ogawa W . Semaglutide once a week in adults with overweight or obesity, with or without type 2 diabetes in an east Asian population (STEP 6): a randomised, double-blind, double-dummy, placebo-controlled, phase 3a trial. Lancet Diabetes Endocrinol. (2022) 10:193–206. doi: 10.1016/S2213-8587(22)00008-0, PMID: 35131037

[5] Chan JM Rimm EB Colditz GA Stampfer MJ Willett WC . Obesity, fat distribution, and weight gain as risk factors for clinical diabetes in men. Diabetes Care. (1994) 17:961–9. doi: 10.2337/diacare.17.9.961, PMID: 7988316

[6] Xu Y Wang L He J Bi Y Li M Wang T . Prevalence and control of diabetes in Chinese adults. JAMA. (2013) 310:948–59. doi: 10.1001/jama.2013.168118, PMID: 24002281

[7] Tobias DK Pan A Jackson CL O'Reilly EJ Ding EL Willett WC . Body-mass index and mortality among adults with incident type 2 diabetes. N Engl J Med. (2014) 370:233–44. doi: 10.1056/NEJMoa1304501, PMID: 24428469 PMC3966911

[8] Flegal KM Carroll MD Ogden CL Curtin LR . Prevalence and trends in obesity among US adults, 1999-2008. JAMA. (2010) 303:235–41. doi: 10.1001/jama.2009.2014, PMID: 20071471

[9] Sturm R . Increases in morbid obesity in the USA: 2000-2005. Public Health. (2007) 121:492–6. doi: 10.1016/j.puhe.2007.01.006, PMID: 17399752 PMC2864630

[10] Melissas J Christodoulakis M Schoretsanitis G Sanidas E Ganotakis E Michaloudis D . Obesity-associated disorders before and after weight reduction by vertical banded gastroplasty in morbidly vs super obese individuals. Obes Surg. (2001) 11:475–81. doi: 10.1381/096089201321209378, PMID: 11501359

[11] Piché M-E Tchernof A Després J-P . Obesity phenotypes, diabetes, and cardiovascular diseases. Circ Res. (2020) 126:1477–500. doi: 10.1161/CIRCRESAHA.120.316101, PMID: 32437302

[12] Hughes TA Gwynne JT Switzer BR Herbst C White G . Effects of caloric restriction and weight loss on glycemic control, insulin release and resistance, and atherosclerotic risk in obese patients with type II diabetes mellitus. Am J Med. (1984) 77 (1):7–17. doi: 10.1016/0002-9343(84)90429-7, PMID: 6377892

[13] National Institutes of Health . Consensus development conference on diet and exercise in non-insulin-dependent diabetes mellitus. Diabetes Care. (1987) 10:639–44. doi: 10.2337/diacare.10.5.639, PMID: 3315516

[14] Nauck MA Meier JJ . The incretin effect in healthy individuals and those with type 2 diabetes: physiology, pathophysiology, and response to therapeutic interventions. Lancet Diabetes Endocrinol. (2016) 4:525–36. doi: 10.1016/S2213-8587(15)00482-9, PMID: 26876794

[15] Drucker DJ Nauck MA . The incretin system: glucagon-like peptide-1 receptor agonists and dipeptidyl peptidase-4 inhibitors in type 2 diabetes. Lancet. (2006) 368:1696–705. doi: 10.1016/S0140-6736(06)69705-5, PMID: 17098089

[16] Gao F Lv X Mo Z Ma J Zhang Q Yang G . Efficacy and safety of polyethylene glycol loxenatide as add-on to metformin in patients with type 2 diabetes: A multicentre, randomized, double-blind, placebo-controlled, phase 3b trial. Diabetes Obes Metab. (2020) 22:2375–83. doi: 10.1111/dom.14163, PMID: 32744358

[17] Shuai Y Yang G Zhang Q Li W Luo Y Ma J . Efficacy and safety of polyethylene glycol loxenatide monotherapy in type 2 diabetes patients: A multicentre, randomized, double-blind, placebo-controlled phase 3a clinical trial. Diabetes Obes Metab. (2021) 23:116–24. doi: 10.1111/dom.14198, PMID: 32965075

[18] Yang G-R Zhao X-L Jin F Shi L-H Yang J-K . Pharmacokinetics and pharmacodynamics of a polyethylene glycol (PEG)-conjugated GLP-receptor agonist once weekly in Chinese patients with type 2 diabetes. J Clin Pharmacol. (2015) 55:152–8. doi: 10.1002/jcph.386, PMID: 25167840

[19] Cai H Chen Q Duan Y Zhao Y Zhang X . Short-term effect of polyethylene glycol loxenatide on weight loss in overweight or obese patients with type 2 diabetes: An open-label, parallel-arm, randomized, metformin-controlled trial. Front Endocrinol (Lausanne). (2023) 14:1106868. doi: 10.3389/fendo.2023.1106868, PMID: 36777344 PMC9909427

[20] Gasbjerg LS Nielsen CK Suppli MP Grøendahl MFG Holst JJ Knop FK . Proglucagon-derived peptides: human physiology and therapeutic potential. Physiol Rev. (2025) 106 (1):529–86. doi: 10.1152/physrev.00057.2024, PMID: 40720286

[21] Moiz A Filion KB Tsoukas MA Yu OHY Peters TM Eisenberg MJ . The expanding role of GLP-1 receptor agonists: a narrative review of current evidence and future directions. EClinicalMedicine. (2025) 86:103363. doi: 10.1016/j.eclinm.2025.103363, PMID: 40727007 PMC12303005

[22] Cao Y Cao S Zhao J Zhao J Zhao Y Liu Y . Efficacy and safety of polyethylene glycol loxenatide in treating mild-to-moderate diabetic kidney disease in type 2 diabetes patients: a randomized, open-label, clinical trial. Front Endocrinol (Lausanne). (2024) 15:1387993. doi: 10.3389/fendo.2024.1387993, PMID: 39099671 PMC11294108

[23] Li J Tian Y Li L Zhao Y Yang S Xu W . Once-weekly glucagon-like peptide receptor agonist polyethylene glycol loxenatide protects against major adverse cardiovascular events in patients with type 2 diabetes: a multicenter ambispective cohort study (FLYING trial). MedComm (2020). (2025) 6:e70094. doi: 10.1002/mco2.70094, PMID: 39949982 PMC11822460

[24] Chandiwana N Barquera S Baur L Buse K Halford J Halpern B . Obesity is a disease: global health policy must catch up. Lancet Glob Health. (2025) 13 (10):e1659–e1660. doi: 10.1016/S2214-109X(25)00275-X, PMID: 40780234

[25] Bray GA Kim KK Wilding JPH . Obesity: a chronic relapsing progressive disease process. A position statement of the World Obesity Federation. Obes Rev. (2017) 18:715–23. doi: 10.1111/obr.12551, PMID: 28489290

[26] Rubino F Cummings DE Eckel RH Cohen RV Wilding JPH Brown WA . Definition and diagnostic criteria of clinical obesity. Lancet Diabetes Endocrinol. (2025) 13:221–62. doi: 10.1016/S2213-8587(24)00316-4, PMID: 39824205 PMC11870235

[27] Nunns M Febrey S Buckland J Abbott R Whear R Bethel A . The quantity, quality and findings of network meta-analyses evaluating the effectiveness of GLP-1 RAs for weight loss: a scoping review. Health Technol Assess. (2025) 1–73. doi: 10.3310/SKHT8119, PMID: 40580049 PMC12336958

[28] Bettencourt-Silva R Neves JS Pedro J Guerreiro V Ferreira MJ Salazar D . Comparative effectiveness of different bariatric procedures in super morbid obesity. Obes Surg. (2019) 29:281–91. doi: 10.1007/s11695-018-3519-y, PMID: 30251091

[29] Kermansaravi M Lainas P Shahmiri SS Yang W Jazi AD Vilallonga R . The first survey addressing patients with BMI over 50: a survey of 789 bariatric surgeons. Surg Endosc. (2022) 36:6170–80. doi: 10.1007/s00464-021-08979-w, PMID: 35064321 PMC9283149

[30] Sun Y Liu B Smith JK Correia MLG Jones DL Zhu Z . Association of preoperative body weight and weight loss with risk of death after bariatric surgery. JAMA Netw Open. (2020) 3:e204803. doi: 10.1001/jamanetworkopen.2020.4803, PMID: 32407504 PMC7225906

[31] Gao W Li Q Zhang R Wu X Suo Q Ji G . The earlier metabolic bariatric surgery is performed, the better expected clinical benefit beyond weight loss: a short-term follow-up study. Eur J Med Res. (2025) 30:615. doi: 10.1186/s40001-025-02885-w, PMID: 40646636 PMC12247318

[32] Fruh SM . Obesity: Risk factors, complications, and strategies for sustainable long-term weight management. J Am Assoc Nurse Pract. (2017) 29 (S1):S3–S14. doi: 10.1002/2327-6924.12510, PMID: 29024553 PMC6088226

[33] Santilli F Simeone PG Guagnano MT Leo M Maccarone MT Di Castelnuovo A . Effects of liraglutide on weight loss, fat distribution, and β-cell function in obese subjects with prediabetes or early type 2 diabetes. Diabetes Care. (2017) 40:1556–64. doi: 10.2337/dc17-0589, PMID: 28912305

[34] Chen X Lv X Yang G Lu D Piao C Zhang X . Polyethylene glycol loxenatide injections added to metformin effectively improve glycemic control and exhibit favorable safety in type 2 diabetic patients. J Diabetes. (2017) 9:158–67. doi: 10.1111/1753-0407.12397, PMID: 26989888

[35] Gentilella R Pechtner V Corcos A Consoli A . Glucagon-like peptide-1 receptor agonists in type 2 diabetes treatment: are they all the same? Diabetes Metab Res Rev. (2019) 35:e3070. doi: 10.1002/dmrr.3070, PMID: 30156747

[36] Pedersen SD Manjoo P Dash S Jain A Pearce N Poddar M . Pharmacotherapy for obesity management in adults: 2025 clinical practice guideline update. CMAJ. (2025) 197:E797–809. doi: 10.1503/cmaj.250502, PMID: 40789597 PMC12350384

[37] Astrup A Carraro R Finer N Harper A Kunesova M Lean MEJ . Safety, tolerability and sustained weight loss over 2 years with the once-daily human GLP-1 analog, liraglutide. Int J Obes (Lond). (2012) 36:843–54. doi: 10.1038/ijo.2011.158, PMID: 21844879 PMC3374073

[38] Mehta A Marso SP Neeland IJ . Liraglutide for weight management: a critical review of the evidence. Obes Sci Pract. (2017) 3 (1):3–14. doi: 10.1002/osp4.84, PMID: 28392927 PMC5358074

