# Supplementary material for: Effect of polyethylene glycol loxenatide on weight loss in super-obese patients with type 2 diabetes: a randomized controlled trial
Source: Front Endocrinol (Lausanne). 2026 Jan 30;17:1689040. doi: 10.3389/fendo.2026.1689040 (PMC12900763; doi:10.3389/fendo.2026.1689040)
Supplement: Supplementary file 1 [file Table1.docx]

Supplementary Material

Table S1

|  | **Mild** | **Moderate** | **Severe*** |
| --- | --- | --- | --- |
| Nausea | Mild discomfort | Noticeable but tolerable sensation | Persistent and intense sensation |
| Vomiting | ≤ 3 times/day | 4-6 times/day | ＞6 times/day |
| Decreased appetite | Marked satiety | Reduced food intake | Significantly reduced food intake |
| Abdominal bloating | Mild sensation of fullness | Abdominal bloating | Abdominal bloating with mild abdominal pain |
| Diarrhea | Slight increase in defecation frequency; stool consistency is loose or soft. | Increased defecation frequency; stool consistency is watery or loose. | Significant increase in defecation frequency; stool consistency is watery. |
| Injection site discomfort | Mild discomfort | Tingling pain | Tingling pain, Redness and swelling |
| Dizziness | Mild discomfort | Clearly noticeable sensation | Recurrent episodes / Intense sensation |
| Headache | Mild discomfort | Clearly noticeable but tolerable sensation | Continuous and intense sensation |
| Fatigue | Mild discomfort | Weakness with activity | Marked fatigue |
| Hypoglycemia | A blood glucose concentration 3.0-3.9 mmol/L. | A blood glucose concentration＜3.0 mmol/L, which is typically the threshold for neuroglycopenic symptoms. | A clinical event characterized by altered mental and/or physical functioning that requires assistance from another person for recovery. |
| **Treatment Measures** | Medical assessment; Enhanced follow-up; Medical intervention based on the medical assessment | | |

Table S1: Grading of Adverse Events and Treatment Measures.

Follow-up of adverse events was conducted using a questionnaire. All adverse reactions reported by study participants were recorded in the case report form. The severity of adverse reactions was analyzed statistically based on the single most severe episode reported by each research participant.

*Including the severity of adverse events leading to the withdrawal of research participants.
